# What is the global prevalence of depression among men who have sex with men? A systematic review and meta-analysis

**DOI:** 10.1186/s12991-022-00414-1

**Published:** 2022-09-12

**Authors:** Elham Nouri, Yousef Moradi, Ghobad Moradi

**Affiliations:** 1 Social Determinant of the Health Research Center, Research Institute for Health Development, Kurdistan University of Medical Sciences, Sanandaj, Iran; 2 Department of Epidemiology and Biostatistics, Faculty of Medicine, Kurdistan University of Medical Sciences, Sanandaj, Iran

**Keywords:** Depression, MSM, Men who have sex with men, Global prevalence, Meta-analysis

## Abstract

**Background:**

Depression due to stigma resulting from their sexual identity, isolation, social exclusion, and insufficient access to care and counseling services has become a health problem among men who have sex with men (MSM).

**Objectives:**

This study aimed to determine the global prevalence of depression among MSM as a systematic review and meta-analysis.

**Methods:**

This study was a systematic review and meta-analysis performed in five steps of search strategy, screening and selecting articles, data extraction, evaluation of the risk of bias, and meta-analysis. In this study, the determined keywords were searched in the databases of PubMed, Scopus, Embase, and Web of Science from January 1913 to July 2021 to find the initial articles, from which data were extracted according to the set checklist in the data extraction stage. Finally, the studies were included in the present meta-analysis according to the inclusion and exclusion criteria, to be evaluated using the Newcastle Ottawa scale checklist. I Square and Q Cochrane were also used to assess the degree of heterogeneity. The analyses were performed using the random-effects model in STATA 16.

**Results:**

The results showed the quality score of the majority of cross-sectional studies included in the meta-analysis (62 studies) was equal to six or seven (moderate), and five ones had a high-quality score. After combining these studies, the pooled prevalence of depression among MSM in the world was 35% (95% CI 31%–39%, I square; 98.95%, *P*-value < 0.001). Population subgroup analysis showed the pooled prevalence of depression among MSM living with HIV was 47% (95% CI 39%-55%, I square; 95.76%, *P*-value < 0.001). Continent subgroup analysis showed the highest pooled prevalence of depression among Asian MSM at 37% (95% CI 31%-43%, I square; 99.07%, *P*-value < 0.001). Also, in the subgroup analysis of the sampling method, the pooled prevalence in the studies which used the respondent-driven sampling method was equal to 34% (95% CI 25%-43%, I square; 99.32%, *P*-value < 0.001). Sensitivity analysis revealed the pooled prevalence of depression in studies included in the meta-analysis was near or around the pooled estimate.

**Conclusion:**

The pooled prevalence of depression among MSM was almost three times higher than the general male population. Therefore, particular and therapeutic interventions such as screening, and harm reduction programs for mental disorders, especially depression, are suggested to be considered in service packages.

## Background

Men who have sex with men (MSM) are marginal and at-risk populations with special and unique health needs [1, 2]. These people also face sexual minority stress caused by constant stress and their sexual orientation, which makes them highly vulnerable to mental health problems [3, 4]. MSM are stigmatized because of their sexual orientation which causes them to avoid expressing their important problems, and become isolated and lonely. They also face discrimination, abuse, lack of social support, and frequent stressful situations. Because of such conditions, they face more problems in receiving health care and are at risk of more psychological complications [1, 5]. These conditions can lead to outcomes such as depression, substance abuse, or feelings of helplessness which limit self-help behaviors [6].

On the other hand, MSM are exposed to high-risk behaviors and its related diseases, such as HIV/AIDS. So, the stigma caused by these conditions can make them suffer from more mental disorders, especially depression [6]. Also, according to the results of previous studies, one of the most critical risk factors for depression is a homosexual orientation which means having sexual and physical tendencies and attraction to the same sex. Research showed gay men were more likely to drug abuse, depression, and suicide [1, 5].

Understanding the prevalence of depression and its associated factors in this population is very important [7]. MSM should be screened for symptoms of depression and anxiety and should seek appropriate mental health services if needed [8–12]. Due to their special conditions, the need for further systematic reviews and meta-analyses on the incidence of depression in them, and the importance of depressive disorders, this study was conducted to determine the global prevalence of depression in MSM as a systematic review and meta-analysis. This meta-analysis is the most up-to-date study in the world to estimate the prevalence of depression in the general population of MSM with appropriate tools and analysis in different subgroups with greater generalizability.

## Methods

The article protocol was registered on the PROSPERO site with the code CRD42021239819.

### Search strategy and screening articles

Articles published from January 1913 to July 2021 in four electronic databases (PubMed, Scopus, Web of Science, and Embase) were retrieved and reviewed. The study main keywords were “Depression” and “Men who Have Sex with Men”. The search syntax is shown in Table 1.

**Table 1:** The search terms and search syntax

Databases	Search syntax
PubMed	("Depressions"[Title/Abstract] OR "Depressive Symptoms"[Title/Abstract] OR "Depressive Symptom"[Title/Abstract] OR ("Symptom"[Title/Abstract] AND "Depressive"[Title/Abstract]) OR ("Symptoms"[Title/Abstract] AND "Depressive"[Title/Abstract]) OR "Emotional Depression"[Title/Abstract] OR ("Depression"[Title/Abstract] AND "Emotional"[Title/Abstract]) OR ("Depressions"[Title/Abstract] AND "Emotional"[Title/Abstract]) OR "Depression"[Title/Abstract] OR "mental health"[Title/Abstract] OR "mental disorder"[Title/Abstract]) AND ("MSM"[Title/Abstract] OR "Men who have sex with men"[Title/Abstract] OR "Homosexual men"[Title/Abstract] OR "Homosexuality"[Title/Abstract] OR "homosexual"[Title/Abstract])
Web of Sciences	TOPIC: (Depressions OR "Depressive Symptoms “OR "Depressive Symptom “OR (Symptom AND Depressive) OR (Symptoms AND Depressive) OR "Emotional Depression” OR (Depression AND Emotional) OR (Depressions AND Emotional) OR "Emotional Depressions” OR depression OR "mental health” OR "mental disorder") AND TOPIC: (MSM OR "Men who have sex with men” OR "Homosexual men” OR "Homosexuality “OR "homosexual")
Embase	(depressions OR 'depressive symptoms' OR 'depressive symptom'/exp OR 'depressive symptom' OR (('symptom'/exp OR symptom) AND depressive) OR (symptoms AND depressive) OR 'emotional depression' OR (('depression'/exp OR depression) AND emotional) OR (depressions AND emotional) OR 'emotional depressions' OR 'depression'/exp OR depression OR 'mental health'/exp OR 'mental health' OR 'mental disorder'/exp OR 'mental disorder') AND (msm:jt OR 'men who have sex with men':jt OR 'homosexual men':jt OR 'homosexuality':jt OR 'homosexual':jt)
Scopus	(TITLE-ABS-KEY (depressions OR “Depressive Symptoms” OR “Depressive Symptom” OR (symptom AND depressive) OR (symptoms AND depressive) OR “Emotional Depression” OR (depression AND emotional) OR (depressions AND emotional) OR “Emotional Depressions” OR depression OR “mental health” OR “mental disorder”) AND TITLE-ABS-KEY (msm OR "Men who have sex with men" OR "Homosexual men" OR "Homosexuality" OR "homosexual"))

Also, to perform gray literature in the present meta-analysis, a manual search was performed by reviewing the references of related articles and the first ten pages of Google Scholar. After retrieving the articles and creating a library in the Endnote software (version nine) for each database, the articles were placed in another library in combination. The duplicate ones were removed based on the default of the Endnote software. Then the remaining articles were reviewed based on their titles, abstracts, and full texts, considering the inclusion criteria. Two authors independently screened the articles based on their titles, abstracts, and full texts, and in case of any disagreement, the results were reviewed by the study supervisor. After screening, the final selection of studies was made by evaluating the full text of selected articles.

### Inclusion and exclusion criteria

This study aimed to determine the global prevalence of depression among MSM. All descriptive and analytical cross-sectional studies were reviewed, and other studies (case studies, cohorts, clinical trials, letters to the editor, case reports, and review studies) were excluded. Articles in languages other than English, and ones which reported the outcome of depression as a mean score with standard deviation and indicators other than percentage or frequency were excluded from the study. In addition, studies with the statistical population of MSM or men who had sex with men were included. These articles were excluded from the study if the statistical population was gay, bisexual, transgender, or other high-risk groups.

### Data extraction

After selecting articles in the screening stage based on their titles, abstracts, and full texts regarding the inclusion criteria, a checklist prepared with the opinion of experts was used to retrieve their information according to the purpose of the study. The checklist components included the author name, study type, publication year, total sample size, country, population type, age, sampling method, depression frequency, continent, and tool for measuring depression.

### Risk of bias

The Newcastle–Ottawa Quality Assessment Scale (NOS) checklist was used to assess the quality of the articles. This checklist is designed to evaluate the quality of cross-sectional studies. Each of these items is given a score of one if observed in the study, and the maximum score for each study is nine points. Scores are ranged from zero stars (the worst case) to nine stars (the best case). Studies with a score of zero to four were categorized as low quality, five to seven as moderate, and more than seven as high quality [13].

### Statistical analysis

According to the study checklist, the total sample size and frequency of depressed MSM were extracted for all studies. Based on the extracted data, the Metaprop command was used to calculate the pooled prevalence, and the results were analyzed [14]. The analysis model was a random effect model. Cochrane Q and I^2^ tests were used to investigate the heterogeneity and variance between the studies selected for meta-analysis [15–18]. To quantitatively determine heterogeneity or as a percentage, the I square index whose range of changes is between zero and 100%, is used. When zero, it indicates the homogeneity of the results, and the larger this value, the greater the heterogeneity between studies. In the Cochrane classification, four levels are considered for heterogeneity: 0–25% (might not be important), 30–60% (may represent moderate heterogeneity), 50–90% (may represent substantial heterogeneity), 75–100% (considerable heterogeneity). Funnel plot and Egger tests were applied to evaluate the publication bias [17, 18]. The aim of this method is to detect and correct the asymmetry of the funnel plot resulting from publication bias Also, trim-and-fill tests were used to determine the effect of publication bias on the estimated pooled prevalence. [19, 20]. Also, the meta-regression analysis and diagram were applied to examine the Association between variables of age and the publication year of selected studies with the estimated pooled prevalence. Subgroup analysis used to find the source of heterogeneity was conducted based on the population type (healthy or HIV infected), age, continent, measuring tools, sampling type, and quality assessment score. The sensitivity analysis was performed by the Metainf command, and statistical analysis using STATA 16.0 while the statistical significance was set at *P* < 0.05.

## Results

### Qualitative results

**Fig. 1:**
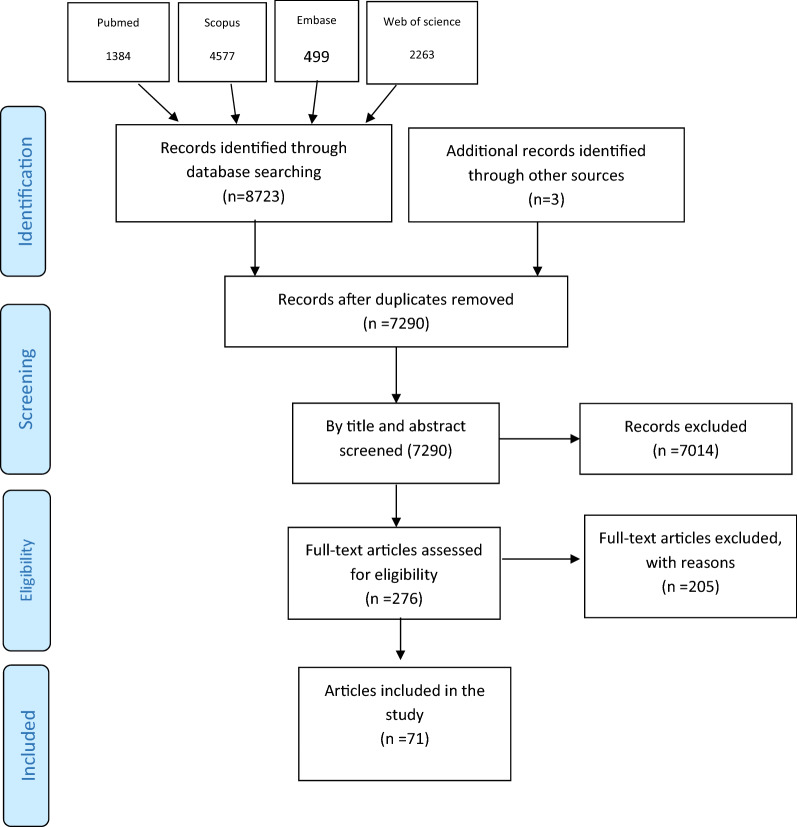
The flowchart of search strategy and syntax

Initially, 8723 articles were obtained from four databases (PubMed, Web of Science, Scopus, and Embase), of which 1384 were from PubMed, 4577 from Scopus, 499 from Embase, and 2263 from the Web of science. After removing similar items in the Endnote software, 7290 articles were selected for screening their titles and abstracts. Then, the full texts of 276 selected studies were reviewed. Finally, 71 studies were included in the analysis [1, 12, 21–89] (Table 2 and Fig. 1), all of which were cross-sectional with the statistical population of MSM. Table 2 shows the lowest mean age (20 years old) was related to the three studies of Bruce et al., Holloway. et al. and Kipke et al. [27, 41, 44] while the highest mean age (57 years old) was related to the study of Zepf et al. [86]. Also, the overall mean age in the meta-analysis was 32 years. In the present meta-analysis, the first study to report the prevalence of depression in MSM was the article of Mills et al. [55], and the most recent study which measured the prevalence of depression in MSM was the article of Clark et al. [32]. The highest number of studies was related to 2018 with 14 articles [26, 31, 34, 46, 47, 50, 58, 60, 62, 69, 71, 77, 84, 89] and 2017 with 11 articles [28, 30, 38, 40, 41, 43, 54, 56, 67, 70, 73]. The smallest sample size was related to the study of Armstrong et al. [25] with 56 people, and the highest was related to the study of Tomori et al. [71] with 11,771 people.

**Table 2:** The characteristics of included articles

Authors [year]	Year	Sample size	Country	Study populations	Age (mean or median)	Method of sampling	Depression	Continents	Measuring tools	NOS score
Ahaneku, H.et al. [2014] [21]	2014	117	Los Angeles	MSM	45	NR	15	America	Beck Depression Inventory II	5
Ahaneku, H.et al. [2016] [22]	2016	205	Tanzania	MSM	25	Respondent-driven sampling	95	Africa	PHQ-9	6
Alvy, L. M.et al. [2011] [23]	2011	1,540	US	MSM	NR	NR	929	America	CES-D	7
An, X.et al. [2020] [24]	2020	334	China	MSM	29	Convenience	116	Asia	CES-D	6
Armstrong, R.et al. [2020] [25]	2020	56	Zambia	MSM	22	Snow-ball	28	Africa	CES-D	7
Brown, M. J.et al. [2018] [26]	2018	337	US	MSM living with HIV	NR	NR	198	America	CES-D	6
Bruce, D.et al.[2014] [27]	2014	200	US	YMSM	20.9	NR	64	America	CES-D	6
Chakrapani, V.et al. [2017] [28]	2017	300	India	MSM	30	Convenience	105	Asia	Beck Depression Inventory	6
Chandler, C. J.et al. [2020] [29]	2020	3294	US	MSM	NR	NR	1278	America	CES-D	6
Chen, Y. H.et al. [2017] [30]	2017	322	California	MSM	NR	NR	42	America	PHQ-9	7
Cherenack, E. M.et al. [2018] [31]	2018	92	US	MSM living with HIV	31	NR	45	America	CES-D	6
Clark, K.et al. [2021] [32]	2021	488	Lebanon	MSM	NR	Respondent-driven sampling	258	Asia	CES-D	8
Deuba, K.et al.[2013] [33]	2013	339	Nepal	MSM	NR	Snow-ball	206	Asia	CES-D	8
Du, M.et al.[2018] [34]	2018	321	China	MSM living with HIV	30	NR	179	Asia	CES-D	6
Dyer, T. P.et al. [2013] [35]	2013	798	US	(BMSMO)	35	NR	319	America	CES-D	7
Fendrich, M.et al. [2013] [36]	2013	177	Chicago	MSM	37	Probability	40	Asia	CES-D	6
Ferro, E. G.et al. [2015] [37]	2015	302	Peru	MSM	32	Convenience	134	America	CES-D	6
Feuillet, P.et al. [2017] [38]	2017	1078	France	MSM living with HIV	NR	NR	334	Europe	CIDI-SF	6
Ha, H. X.et al.[2014] [39]	2014	451	Vietnam	MSM	30	Respondent-driven sampling	249	Asia	CES-D	6
Holloway, I. W.et al. [2017] [40]	2017	150	California	MSM living with HIV	45	NR	96	America	CES-D	7
Holloway, I. W.et al. [2017] [41]	2017	526	California	YMSM	20	Stratified	52	America	CES-D	8
Hu, Y.et al.[2019] [42]	2019	1518	China	MSMO	27	Non-probability sampling	534	Asia	CES-D	6
Hylton, E.et al. [2017] [43]	2017	1376	Russia	MSM	30	Respondent-driven sampling	505	Europe	CES-D	7
Kipke, M. D.et al. [2007] [44]	2007	526	US	yMSM	20	Venue-based probability sampling	110	America	CES-D	7
Klein, H.et al.[2014] [45]	2014	332	US	MSM	43	Random	86	America	CES-D	7
Kunzweiler, C. P.et al. [2018] [46]	2018	711	Kenya	MSM	24	Respondent-driven sampling	81	Africa	PHQ	7
Levine, E. C.et al. [2018] [47]	2018	176	New York	MSM	34	Stratified	120	America	CES-D	7
Li, J.et al. [2016] [48]	2016	321	China	MSM living with HIV	30	NR	179	Asia	CES-D	6
Li, R.et al. [2016][49]	2016	547	China	MSM	30	NR	169	Asia	CES-D	5
Liu, Y.et al. [2018][50]	2018	807	China	MSM	NR	Respondent-driven sampling	267	Asia	SDS	6
Maragh-Bass, A. C.et al. [2020] [51]	2020	357	US	MSM	48	NR	180	America	PHQ	7
Mayer, K. H.et al. [2014] [52]	2014	1553	US	MSM	40	NR	698	America	CES-D	7
Mayer, K. H.et al. [2015] [53]	2015	307	India	MSM	30	NR	67	Asia	CES-D	6
Mgopa, L. R.et al. [2017] [54]	2017	345	Tanzania	MSM	31	Driven sampling technique	245	Africa	PHQ	6
Mills, T. C.et al. [2004] [55]	2004	2,678	US	MSM	NR	Household-based probability sample	461	America	CES-D	7
Miltz, A. R.et al. [2017] [56]	2017	1340	United Kingdom	MSM	NR	NR	166	Europe	PHQ	6
Mimiaga, M. J.et al. [2013] [57]	2013	150	India	MSM	25	NR	43	Asia	MINI	6
Mo, P. K.et al. [2018] [58]	2018	225	China	MSM	32.2	NR	109	Asia	DASS	7
Mu, H.et al. [2016] [59]	2016	807	China	MSM	NR	Respondent-driven sampling	55	Asia	DSM	5
Murphy, Patrick.et al. [2018] [60]	2018	278	United Kingdom and Ireland	MSM living with HIV	44	NR	161	Europe	HADS	6
O'Cleirigh, C.et al. [2009] [61]	2009	503	New England	MSM	42	NR	43	Europe	PHQ	6
Pan, X.et al. [2018] [62]	2018	454	China	MSM	33	Respondent-driven sampling	157	Asia	CES-D	6
Parker, R. D.et al. [2015] [63]	2015	265	Estonia	MSM	32	Random	34	Europe	EST-Q	7
Peng, L.et al. [2020] [64]	2020	578	China	MSM	28	Convenience	189	Asia	CES-D	6
Prabhu, S.et al.[2020] [65]	2020	1454	India	MSM	37	Respondent-driven sampling	241	Asia	PHQ	6
Reisner, S. L.et al. [2009] [66]	2009	197	Massachusetts	MSM	38	Respondent-driven sampling	65	America	CES-D	7
Rüütel, K.et al. [2017] [67]	2017	265	Estonia	1235)	32	NR	84	Europe	EST-Q	7
Safren, S. A.et al. [2009] [12]	2009	210	India	MSM	28	NR	115	Asia	CES-D	6
Secor, A. M.et al. [2014] [1]	2014	112	Kenya	MSM	26	NR	18	Africa	PHQ	7
Sivasubramanian, M.et al. [2011] [68]	2011	150	India	MSM	25	NR	43	Asia	DSM	7
Su, X.et al.[2018] [69]	2018	507	China	MSM	NR	Convenience	136	Asia	CES-D	6
Tao, J.et al. [2017] [70]	2017	364	China	MSM living with HIV	28	NR	131	Asia	HADS	7
Tomori, C.et al. [2018] [71]	2018	11,771	India	MSM	26	Respondent-driven sampling	1502	Asia	PHQ	7
Wagner, G. J.et al. [2019] [72]	2019	226	Beirut	YMSM	24	NR	36	Asia	PHQ	7
Wang, Y.et al. [2017] [73]	2017	547	China	MSM	30	NR	285	Asia	CES-D	6
Wei, D.et al. [2020] [74]	2020	578	China	MSM	NR	NR	208	Asia	CES-D	5
Wendi, D.et al. [2016] [75]	2016	316	Lesotho	MSM	23	Respondent-driven sampling	69	Africa	PHQ	8
White, J. J.et al. [2020] [76]	2020	256	US	MSM	39	NR	96	America	CES-D	6
Wilkerson, J. M.et al. [2018] [77]	2018	421	India	MSM	NR	Respondent-driven sampling	242	Asia	CES-D	7
Williams, J. K.et al. [2015] [78]	2015	1522	US	MSM	NR	NR	615	America	CES-D	7
Wim, V. B.et al. [2014] [79]	2014	591	Belgium	Men who have sex with men	34	NR	171	Europe	CES-D	6
Wu, Y.et al. [2015] [80]	2015	184	China	MSM living with HIV	31	NR	79	Asia	CES-D	6
Yan, H.et al. [2014] [81]	2014	204	China	MSM	NR	Respondent-driven sampling	94	Asia	CES-D	6
Yan, H.et al. [2019] [82]	2019	347	China	MSM living with HIV	34	Convenience	134	Asia	CES-D	6
Yang, C.et al. [2013] [83]	2013	188	Baltimore	MSM	38	Random	35	America	CES-D	7
Yu, L.et al. [2018] [84]	2018	807	China	MSM	NR	Respondent‐driven sampling	267	Asia	SDS	6
Zeng, X.et al. [2016] [85]	2016	1235	China	MSM	31.6	Non-probability	563	Asia	CES-D	7
Zepf, R.et al. [2020] [86]	2020	281	San Francisco	MSM living with HIV	57	NR	77	America	PHQ	8
Zhang, S.et al. [2019] [87]	2019	547	China	MSM	30	Snowball	169	Asia	CES-D	6
Zhao, Y.et al. [2020] [88]	2020	338	Malawi	MSM	25	Respondent-driven sampling	102	Africa	NR	6
Zhu,Y.et al. [2018] [89]	2018	342	China	MSM	28	Convenience	153	Asia	GHQ	7

### Quality assessment (risk of bias)

As shown in Table 1, the risk of bias in the study results ranged from five to eight. The quality score of four cross-sectional studies included in the meta-analysis was five. It was six, seven, and eight for 35, 27, and five studies. Most of the studies included in the meta-analysis had good quality for the analysis (Table 2).

### Quantitative results

#### Prevalence of depression among MSM:

**Fig. 2:**
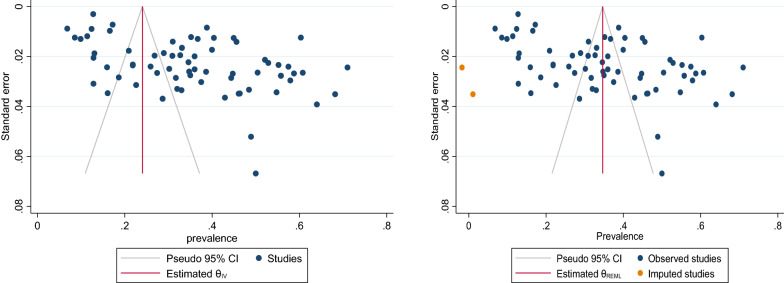
Funnel plot/trim-and-fill test of the pooled prevalence of depression in MSM population

**Fig. 3:**
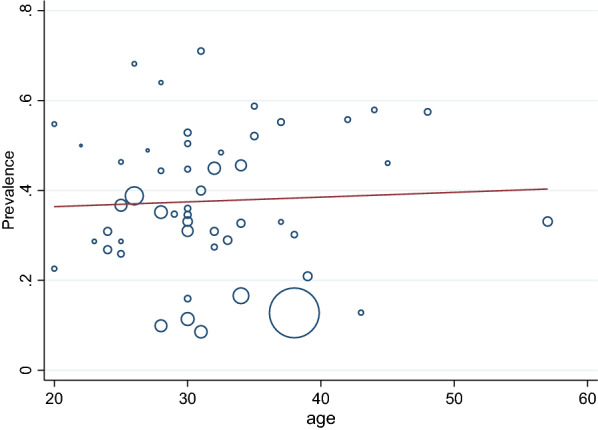
The meta-regression analysis of the effect of age on the pooled prevalence of depression in MSM

**Fig. 4:**
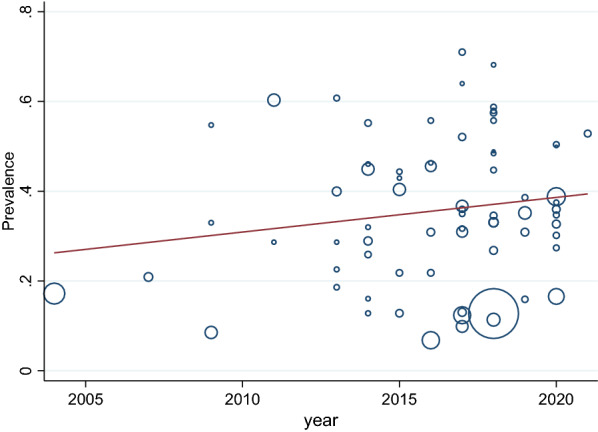
The meta-regression analysis of the effect of year on the pooled prevalence of depression in MSM

The sample size of MSM in a total of 71 articles was 51,541 people, of whom 15,171 had depressive symptoms. After combining these studies, the pooled prevalence of depression in MSM was 35% (95% CI 31–39%, I^2^: 98.95%). The prevalence range in the studies varied from 7 to 71%, with the lowest prevalence equal to 7% (95% CI 5–9%) related to the study of Mu, H. et al. [59], and the highest prevalence equal to 71% (95% CI 66–76%) related to the study of Mgopa L.R. et al. [54] (Table 3). The Eggers test results showed publication bias in calculating the pooled prevalence of depression in MSM (*B* = 10.71, SE = 0.197, *P* < 0.001). To show the publication bias, the funnel plot diagram (Fig. 2) was used. The trim-and-fill test showed publication bias had no considerable effect on the final overall estimate (*P* = 0.347, CI = 0.308—0.385) (Fig. 2). Meta-regression analysis was also used to investigate the association between the age of MSM and the publication year of the studies included in the meta-analysis, the results of which are shown in Figs. 3 and 4

**Table 3:** The pooled prevalence of depression among MSM (over all prevalence, subgroup analysis of depression)

Depression	No. of study(ss)	No. of depression	Pooled prevalence	Heterogeneity assessment		Z Score (*P* Value)
				I2	*p*-value	
Overall	71 (51541)	15171	% 35 (% 31 - % 39)	% 98.95	<0.00	
Population						
Healthy	60 (47788)	13558	% 33 (%28 - %37)	% 99.02	<0.00	9.21 (0.001)
HIV	11 (3753)	1613	% 47 (%39 - %55)	% 95.76	<0.00	
Continent						
Asia	33 (27841)	7280	% 37 (% 31 - % 43)	% 99.07	<0.00	2.96 (0.400)
Africa	7 (2083)	638	% 34 (%17 - % 53)	% 98.69	<0.00	
America	23 (15921)	5755	% 35 (% 29 - % 42)	% 98.68	<0.00	
Europe	8 (5696)	1498	%26 (%17 - %37)	% 98.65	<0.00	
Age						
≥30	28 (23350)	5388	%33 (%26 - %39)	% 98.84	<0.00	0.79 (0.001)
<30	26 (11122)	4027	%37 (%30 - %43)	% 98.01	<0.00	
Not	17 (17069)	5756	%36 (%27 - %44)	%99.25	<0.00	
Measuring tools						
DASS	1 (225)	109	%48 (%42 - %55)	–	–	458.26 (0.001)
PHQ	13 (17943)	2795	%23 (%16 - %31)	% 98.74	<0.00	
ESTQ	2 (530)	118	%22 (%18 - %25)	0.00	<0.00	
MINI	1 (150)	43	%29 (%22 - %37)	–	–	
DSM	2 (957)	98	%9 (%8 - %11)	0.00	<0.00	
HADS	2 (642)	292	%45 (%42 - %49)	0.00	<0.00	
CES-D	43 (27305)	10473	%40 (%36 - %44)	% 97.99	<0.00	
SDS	2 (1614)	534	%33 (%31 - %35)	0.00	<0.00	
CIDI	1 (1078)	334	%31 (%28 - %340	–	–	
GHQ	1 (342)	153	%45 (%39 - %50)	–	–	
Becks depression inventory	2 (417)	120	%28 (%24 - %32)	0.00	<0.00	
Not	1 (338)	102	%30 (%25 - %35)	–	–	
Method of sampling						
Driven sampling technique	17 (21152)	4494	%34 (%25 - %43)	% 99.32	<0.00	10.98 (0.030)
Convenient	7 (2710)	967	%36 (%32 - %41)	%86.20	<0.00	
Non-probability	5 (3695)	1500	%44 (%35 - %53)	% 96.42	<0.00	
Probability sample	8 (4868)	938	%23 (%15 - %32)	% 97.21	<0.00	
Not	34 (19116)	7272	%36 (%31 - %42)	% 98.18	<0.00	
Score of NOS						
SCORE=5	4 (2049)	447	%20 (%7 - %39)	% 98.76	<0.00	3.56 (0.31)
SCORE=6	35 (19684)	6777	%37(%33 - %42)	%97.94	<0.00	
SCORE=7	27 (27858)	7285	%34(%27 - %42)	%99.32	<0.00	
sSCORE=8	5 (1950)	662	%33(%15 - %55)	%98.97	<0.00

##### Subgroup analysis

In this study, subgroup analysis was performed based on the population type, continent, age of MSM, measuring tools, and sampling type; the results are shown in Table 3.

##### Subgroup analysis based on the population type

The subgroup analysis results for the population type showed the sample size of MSM living with HIV in 11 studies was 3753 individuals, and the pooled prevalence of depression was 47% (95% CI 39–55%, I^2^: 95.76%). Also, in 60 studies with a sample size of 47,788 healthy MSM without HIV, the prevalence of depression was 33% (95% CI 28–37%, I^2^: 99.02%) (Table 3). Heterogeneity in the population type subgroup was at the considerable level. The observed difference between the prevalence of depression in the MSM population with HIV and healthy ones was statistically significant (Table 3).

##### Subgroup analysis based on the continent

The results of the subgroup analysis based on the continent showed 33 studies were conducted in Asia with a sample size of 27,841 MSM, according to the results of which 7280 of these people were suffering from depression. The pooled prevalence in Asian MSM was 37% (95% CI 31–43%, I^2^: 99.07%). In addition, eight studies were conducted in Europe with a sample size of 5696 MSM, of whom 1498 had depression. The pooled prevalence in European MSM was 26% (95% CI 17–37%, I^2^: 98.65%). There were seven studies in Africa with a sample size of 2083 MSM; according to the results, 638 people were depressed. The meta-analysis results showed the pooled prevalence in African MSM was 34% (95% CI 17–53%, I^2^: 98.69%). Finally, 23 studies were performed in the Americas, the results of which showed out of 15,921 MSM participants, 5755 had depression, and the pooled prevalence in the present meta-analysis was 35% (95% CI 29–42%, I^2^: 98.68%). Asia had the highest prevalence of depression (Table 3). Heterogeneity in the continent subgroup was at the considerable level. The difference between the geographical areas in terms of the prevalence of depression was not statistically significant (Table 3).

##### Subgroup analysis based on age

The results of subgroup analysis based on the age showed 28 studies had a sample size of 23,350 MSM aged 30 and less than 30 years, and out of the total participants, 5388 people had depression. The pooled prevalence of depression in MSM aged 30 and less than 30 years was 33% (95% CI 26–39%, I^2^: 98.84%). The total sample size of 26 studies was 11,122 MSM older than 30 years, of whom 4027 were depressed, and the pooled prevalence of depression was 37% (95% CI 30–43%, I^2^: 98.01%) (Table 3). Heterogeneity in the age subgroup was at the considerable level. The difference in the prevalence of depression between MSM aged more and less than 30 years was statistically significant (Table 3).

##### Subgroup analysis based on the measurement tool

After combining the studies which used CES-D tools, the pooled prevalence of depression was 40% (95% CI 36–44%, I^2^:97.99%). Also, by combining the studies which used PHQ tools to diagnose depression, the pooled prevalence was 23% (95% CI 16–31%, I^2^: 98.74%). The present study showed the prevalence of depression was higher in studies which used the CES-D tool (Table 3). Heterogeneity in the measurement tool subgroup was at the considerable level. The results showed the differences in the prevalence of depression in MSM based on the different diagnostic tools were statistically significant (Table 3).

##### Subgroup analysis based on the sampling method

Seventeen articles with a sample size of 21,152 MSM, of whom 4494 were depressed, used the driven sampling method and showed a pooled prevalence of 34% (95% CI 25–43%, I^2^:99.32%). Also, after combining the studies which used the conventional method, the pooled prevalence was 36% (95% CI 32–41%, I^2^:86.20%). Five articles with a pooled prevalence of 44% (95% CI 35–53%, I^2^: 96.42%) used the non-probability sampling method. Also, eight studies with a pooled prevalence of 23% (95% CI 15–32%, I^2^: 97.21%) applied probability sampling (Table 3). Heterogeneity in the sampling type subgroup was at a considerable level. The observed differences in the prevalence of depression in the MSM community based on the sampling method of the initial studies were statistically significant (Table 3).

### Sensitivity analysis

Sensitivity analysis in this study was performed to investigate the effect of separate removal of the studies included in the meta-analysis on the outcome of the prevalence of depression in MSM. Its results are shown in Table 3. Each of 13 studies out of the total number of ones included in the meta-analysis, if separately removed from the final analysis, the final pooled estimate would increase from 35% to nearly 36% in the present meta-analysis. However, the rest of the studies did not change the overall pooled estimate if omitted. This confirmed the overall result of the present meta-analysis and its high accuracy so that a large number of selected studies, if considered or not in the analysis, did not make any significant changes in the final pooled estimate (Table 4).

**Table 4:** Sensitivity analysis for the global prevalence of depression among MSM

Study omitted	Coef	[95% Conf. Interval]
Mo et al. [2018]	0.354	0.316–0.392
Mgopa et al. [2017]	0.351	0.313–0.388
Mills et al. [2004]	0.359	0.319–0.398
Mimiaga et al. [2013]	0.357	0.318–0.395
Mu et al. [2016]	0.360	0.322–0.398
Murphy Patrick.et al. [2018]	0.353	0.314–0.391
Ahaneku et al. [2016]	0.354	0.316–0.393
Alvy et al. [2011]	0.352	0.315–0.389
An et al. [2020]	0.356	0.317–0.394
Armstrong et al. [2020]	0.354	0.316–0.392
Brown et al. [2018]	0.352	0.314–0.391
Bruce et al. [2014]	0.356	0.318–0.395
Chakrapani et al. [2017]	0.356	0.317–0.394
Chen et al. [2017]	0.359	0.321–0.398
Clark et al. [2021]	0.353	0.315–0.391
Deuba et al. [2013]	0.352	0.314–0.390
Miltz et al. [2017]	0.359	0.320–0.398
Wei et al. [2020]	0.356	0.317–0.394
Rtel et al. [2017]	0.356	0.318–0.395
Yan et al. [2019]	0.355	0.317–0.394
Zepf et al. [2020]	0.357	0.318–0.395
Zeng et al. [2016]	0.354	0.316–0.393
Yu et al. [2018]	0.356	0.318–0.395
Yang et al. [2013]	0.358	0.320–0.397
Wu et al. [2015]	0.355	0.316–0.393
Zhao et al. [2020]	0.357	0.318–0.395
Dyer et al. [2013]	0.355	0.317–0.394
Hylton et al. [2017]	0.356	0.317–0.394
Wang et al. [2017]	0.353	0.315–0.392
Tao et al. [2017]	0.356	0.317–0.394
Pan et al. [2018]	0.356	0.317–0.394
Hu et al. [2019]	0.356	0.317–0.395
Wim et al. [2014]	0.357	0.318–0.395
Cherenack et al. [2018]	0.354	0.316–0.392
Prabhu et al. [2020]	0.359	0.320–0.398
O'Cleirigh et al. [2009]	0.360	0.321–0.398
Ha et al. [2014]	0.353	0.315–0.391
Du et al. [2018]	0.353	0.315–0.391
Feuillet et al. [2017]	0.356	0.318–0.395
Kunzweiler et al. [2018]	0.359	0.321–0.398
Li et al. [2016]	0.353	0.315–0.391
Li R.et al. [2016]	0.356	0.318–0.395
Liu et al. [2018]	0.356	0.318–0.395
Maragh-Bass et al. [2020]	0.354	0.315–0.392
Williams et al. [2015]	0.355	0.317–0.394
Wilkerson et al. [2018]	0.353	0.315–0.391
White et al. [2020]	0.356	0.317–0.394
Wendi et al. [2016]	0.358	0.319–0.396
Kipke et al. [2007]	0.358	0.319–0.397
Sivasubramanian et al. [2011]	0.357	0.318–0.395
Fendrich et al. [2013]	0.358	0.319–0.396
Klein et al. [2014]	0.357	0.319–0.396
Reisner et al. [2009]	0.356	0.318–0.395
Ahaneku et al. [2014]	0.359	0.321–0.397
Tomori et al. [2018]	0.359	0.321–0.397
Yan et al. [2014]	0.354	0.316–0.393
Chandler et al. [2020]	0.355	0.317–0.394
Mayer et al. [2015]	0.358	0.319–0.396
Secor et al. [2014]	0.359	0.320–0.397
Wagner et al. [2019]	0.359	0.320–0.397
Levine et al. [2018]	0.351	0.313–0.389
Su et al. [2018]	0.357	0.318–0.396
Peng et al. [2020]	0.356	0.318–0.395
Parker.et al. [2015]	0.359	0.321–0.398
Holloway et al. [2017]	0.360	0.321–0.398
Zhang et al. [2019]	0.356	0.318–0.395
Safren et al. [2009]	0.353	0.315–0.391
Zhu et al. [2018]	0.354	0.316–0.393
Ferro et al. [2015]	0.355	0.316–0.393
Holloway et al. [2017]	0.352	0.314–0.390
Mayer et al. [2014]	0.354	0.316–0.393

## Discussion

The present meta-analysis showed depression had a significant prevalence among MSM populations worldwide. The MSM community and other sexual minorities suffer from depression due to rejection by families and others, as well as increased discrimination preventing them from accessing health services. Therefore, screening programs are essential for early diagnosis of mental disorders in these communities. According to previous studies, the determinants of depression in MSM communities include HIV-related stigma, unemployment, sleep disorders, smoking, racism against blacks, birth abroad, initiation of ART, and lack of access to mental health care. Aging, internalized stigma, and lack of self-efficacy, and social support are also important in this complication [55, 90, 91]. However, the prevalence of depression in MSM and other key populations can be influenced by the factors mentioned in the study of Mohamad Faisal et al. [90]. In this study, various factors were mentioned to be effective in increasing the incidence or prevalence of depression in the MSM community, the most important of which was their infection with infectious diseases, especially HIV/AIDS. Fear of stigma due to HIV/AIDS and the decline in referrals for prevention and supportive care services exacerbate their loneliness and isolation. Isolation from the society predisposes MSM to mental disorders, especially depression. Infection with HIV/AIDS and being a sexual minority are two critical factors exacerbating depressive symptoms in MSM.

The pooled prevalence of depression was 35% in this meta-analysis, confirming the high prevalence of depression in MSM communities compared to the general population whose depression prevalence, according to the previous studies, was reported at 13% [92], 14.4% [93], 11.3% [94], and 4.4% [95]. In other high-risk groups like female sex workers (FSWs), the depression prevalence was higher than in the MSM population [96, 97] because the women were more vulnerable than men for several reasons, including factors of biological origins, differences in physical strength, and personality traits [98].

All sexual minorities, especially MSM, have insufficient social support and experience more mental disorders, especially depression. The results of previous studies showed improving social support and its components could lead to reducing depressive symptoms among MSM [99]. In the studies of Shao Bing et al. [100], and Huamei Yan [82], the results showed lack of social support and rejection by families and friends were critical and significant factors in the development of mental disorders, especially depression, in the MSM community. To confirm this association, studies showed if there were adequate and appropriate family or community support for people of sexual minorities, especially MSM, the risk of mental disorders such as depression, anxiety, or suicide would reduce [2, 8, 101]. Also, in 69 low- or middle-income countries, sexual minorities, especially MSM, are considered criminal. So, these communities are exposed to severe stigma and discrimination [102, 103].

If the MSM community suffers from acute or stigmatized diseases or infections such as HIV/AIDS or other sexually transmitted diseases, the incidence or chance of developing mental disorders, especially depression, will be multiplied. In line with this hypothesis, the result of the subgroup analysis showed depression prevalence in MSM living with AIDS/HIV (47%) was higher than that in healthy ones. A meta-analysis conducted to determine the prevalence of HIV/AIDS in the MSM community in 2020 reported a prevalence of 43% [104]. One of the reasons for the increase in depression in MSM living with HIV/AIDS compared to healthy ones is the decrease in the number of their sexual partners after disclosing HIV/AIDS [105]. Following the decline in the number of their partners, MSM living with HIV are also losing their peers' support. In this case, these people are prone to depression and even suicide. On the other hand, in addition to stigma, discrimination, and lack of social support from families, friends, and the community due to their sexual identity, MSM also faces the stigma associated with HIV/AIDS, increasing depression in this group compared to healthy MSM or those without HIV [22, 66].

The results showed depression was more prevalent among MSM aged more than 30 years than younger ones. Older MSM experience stigma, discrimination, and lack of social support from families. They also face high-stress levels leading to mental disorders such as depression [106]. Geographically, this meta-analysis showed Asian MSM had a higher depression prevalence than those living in other continents. Asians are more prone to depression and other mental disorders due to different cultures, how they deal with homosexuality, rejection by families and friends, and social isolation [107].

On the contrary, in Europe, due to the acceptance of the sexual identity of sexual minorities, especially MSM, and treating them better, they are less likely to suffer from depression and mental disorders. In the initial studies, various tools were used to measure the prevalence of depression in the MSM community, the most widely used of which was CES-D. Therefore, in subgroup analysis, after combining the results of these initial studies, the prevalence was higher than that of studies which used other measurement tools. It can be noted that the CES-D tool has become increasingly popular among researchers for measuring and reporting depression prevalence in the MSM community.

Subgroup analysis based on the checklist of different NOS scores in this study and the results in Table 3 showed the depression prevalence in MSM was 20% with a confidence interval of 7% to 39% after combining the results of cross-sectional studies with a score of five. In contrast, after combining the results of cross-sectional studies with a high score of eight, this prevalence was 33% with a confidence interval of 15% to 55%. This indicates an underestimation of the depression prevalence in MSM in low-quality cross-sectional studies. The highest depression prevalence in the MSM community was related to several studies with the quality scores of six and seven. Also, more studies in these two categories created a narrower confidence interval than those in the other two categories (i.e., the scores five and eight).

In this study, heterogeneity was high, and the authors decided to perform a subgroup analysis based on the essential variables such as the population type, depression diagnosis tools, geographical areas, sampling methods, age, and finally, different scores of quality evaluation. As can be seen in Table 3, heterogeneity decreased in some classes, but it was not justifiable due to the small number of articles. Therefore, it can be concluded the mentioned and intended variables for subgroup analysis cannot be considered as the heterogeneity factor in the final results of the present meta-analysis because they have not reduced heterogeneity in the subgroup analysis. Finally, other variables and factors unfortunately not considered in the initial studies selected for the meta-analysis can cause fundamental differences between the selected studies and increase heterogeneity. So, we could not perform subgroup analysis based on these variables or factors.

## Limitations

One of the limitations of this meta-analysis was the lack of reporting the mean age in many initial studies. On the other hand, because this meta-analysis aimed to determine the prevalence, and only cross-sectional studies were used, after combining these articles, heterogeneity was high. This can be considered as one of the main limitations of prevalence meta-analyses. In addition, this study tried to identify the sources of heterogeneity using subgroup analysis, but due to the lack of reporting other essential variables such as the number of sexual partners, having or not having social relationships and family supports, the place where they live, and living with family or single, identifying these sources was not possible. Also, in the initial studies, the diseases to which MSM people are exposed have not been reported. So, subgroup analysis was not possible based on those variables to compare the prevalence of depression in different groups of MSM. Due to the lack of reporting these variables in the initial studies, and the lack of accurate identification of the heterogeneity sources in the present meta-analysis, the results should be prudently considered, and more detailed studies with appropriate sample sizes are needed to determine the exact prevalence of depression in this community.

## Strengths

In the present meta-analysis, the prevalence of depression in all the MSM population was studied and analyzed for the first time with an impressive number of preliminary studies. Finally, based on the results of the present meta-analysis, the prevalence of depression in the MSM community seems to be increasing which can be considered a warning. This meta-analysis also confirmed the need to design and provide a mental health package, especially for depression, when providing services to these people. This package of mental health services can include screening, treatment, care, and follow-up programs.

## Conclusion

The pooled prevalence of depression in the at-risk group of MSM was approximately three times higher than that of the general population. Therefore, it is necessary to pay special attention to screening MSM and to plan some interventions such as treatment of mental disorders, especially depression. Special preventive measures and interventions are needed to better treat and manage psychological problems such as depression in MSM, especially at younger ages. Also, creating a supportive and friendly culture in the general population toward MSM reduces their isolation, rejection, and the probability of depression.

## Data Availability

The data extracted for analyses are available from the corresponding author upon reasonable requests.

## Abbreviations

MSM: Men who have sex with men
BDI: The Beck Depression Inventory
PHQ: The Patient Health Questionnaire
CES-D: The Center for Epidemiology Studies Depression
HADS: Hospital Anxiety and Depression Scale
DASS: Depression Anxiety Stress Scales
NOS: New Castle–Ottawa Quality Assessment
